# Design and Implementation of a Specialised Millimetre-Wave Exposure System for Investigating the Radiation Effects of 5G and Future Technologies

**DOI:** 10.3390/s24051516

**Published:** 2024-02-26

**Authors:** Negin Foroughimehr, Andrew Wood, Ray McKenzie, Ken Karipidis, Ali Yavari

**Affiliations:** 1 6G Research and Innovation Lab, Swinburne University of Technology, Melbourne, VIC 3122, Australia; nforoughimehr@swin.edu.au (N.F.); awood@swin.edu.au (A.W.); rmckenzie@swin.edu.au (R.M.); kkaripidis@swin.edu.au (K.K.); 2 School of Science, Computing and Engineering Technologies, Swinburne University of Technology, Melbourne, VIC 3122, Australia; 3 School of Health Sciences, Swinburne University of Technology, Melbourne, VIC 3122, Australia; 4 Australian Radiation Protection and Nuclear Safety Agency (ARPANSA), Melbourne, VIC 3085, Australia

**Keywords:** mmWave, non-ionising radiation, radiation protection, 5G, RF exposure system

## Abstract

As the fifth-generation (5G) network is introduced in the millimetre-wave (mmWave) spectrum, and the widespread deployment of 5G standalone (SA) is approaching, it becomes essential to establish scientifically grounded exposure limits in the mmWave frequency band. To achieve this, conducting experiments at specific frequencies is crucial for obtaining reliable evidence of potential biological impacts. However, there is a literature gap where experimental research either does not utilise the mmWave high band (e.g., the 26 Gigahertz (GHz) band) or most studies mainly rely on computational approaches. Moreover, some experimental studies do not establish reproducible test environment and exposure systems. Addressing these gaps is vital for a comprehensive exploration of the biological implications associated with mmWave exposure. This study was designed to develop and implement a mmWave exposure system operating at 26 GHz. The step-by-step design and development of the system are explained. This specialised system was designed and implemented within an anechoic chamber to minimise external electromagnetic (EM) interference, creating a controlled and reproducible environment for experiments involving high-frequency EM fields. The exposure system features a 1 cm radiation spot size, enabling highly localised exposure for various biological studies. This configuration facilitates numerous dosimetry studies related to mmWave frequencies.

## 1. Introduction

Global fifth-generation (5G) subscriptions are predicted to exceed 5.3 billion by 2029, accounting for 58 percent of all mobile subscriptions worldwide at that point in time [1]. The telecommunications industry, driven by a constant demand for innovation, witnesses the regular emergence of new technologies and devices. Over the years, five generations of mobile communication networks have evolved, primarily operating within frequencies below 6 Gigahertz (GHz) [2]. However, the current 5G technology marks a shift, incorporating higher frequencies to meet the demands of applications and services, such as high-resolution video streaming, telepresence, and virtual reality (VR).

Fifth-generation technology offers data transfer speeds of up to 10 gigabits per second (Gb/s) and a latency of less than one millisecond (ms), which specifically involves using millimetre-wave (mmWave) frequency bands, such as 26 GHz and beyond, extending spectrum utilisation beyond the sub-6 GHz band [3,4]. As 5G evolves, the requirements for total transmitted traffic within a cell and user-specific bit rates continually rise. This necessitates exploring a new spectrum (e.g., mmWave), innovative licensing methods, and sophisticated coordination of both high- and low-frequency bands. These efforts are crucial for delivering high capacity, improved throughput, and widespread coverage [2].

Initially recognised as sub-6 GHz frequency bands (450–6000 MHz), Frequency Range 1 (FR1) has shifted to (410–7125 MHz), also known as the mid-band/low-band [5]. Earlier wireless communication standards traditionally used some of these bands, such as Long-Term Evolution (LTE) band 46 (5150–5925 Megahertz (MHz)). On the other hand, Frequency Range 2 (FR2) (24,250–52,600 MHz) has a short electrical wavelength, resulting in a shorter transmission range but a wider operating bandwidth compared to FR1. The mmWave region is currently deployed for 5G higher bands and is anticipated to play a substantial role in the forthcoming sixth-generation (6G) mobile networks. The mmWave region falls within the radiofrequency (RF) region of the electromagnetic (EM) spectrum that spans between 30 and 300 GHz, corresponding to a wavelength range of 10 to 1.0 mm. However, the spectrum around 20–30 GHz is also commonly referred to as mmWave [2].

While FR1 has been used in mobile networks since the inception of the first generation nearly four decades ago, extensive research has been conducted to understand its radiation effects. With the anticipation of deploying the 5G spectrum in the FR2 region across numerous countries to facilitate diverse applications [5], exploring its potential health effects is critical. This paper offers a comprehensive overview of the design, development, and performance testing of our proposed well-characterised exposure system facilitating experiments on various samples (including biological samples) exposed to RF-EM fields associated with 5G mobile broadband network technologies.

### 1.1. Fifth-Generation in Australia

The Australian Communications and Media Authority (ACMA) regulates communication and media services in Australia. The operation of 5G involves different frequency bands in Australia as described by ACMA as follows:

- Low-band 5G, which uses frequency bands below 1 GHz, provides longer ranges and better penetration into buildings but sacrifices speed and capacity.
- Mid-band 5G, operating between 1 and 6 GHz bands, offers a balance between range, building penetration, and network speed.
- mmWave band (high-band) 5G, using frequencies at 26 GHz band (25.1 GHz–27.0 GHz) and above, delivers faster speeds and higher capacity but with shorter range and less penetration.

In Australia, 4G and 5G wireless broadband services have been active since 2016 and 2019 in the 3.4 GHz and 3.6 GHz bands, respectively. Aircraft radio altimeters, on the other hand, operate above 4.2 GHz. Australia officially rolled out 5G services in late 2018, predominantly in the mid-band spectrum ranging from 3.575 to 3.7 GHz. The deployment of the mmWave band 5G started in select cities in mid-2021, and spectrum licenses were auctioned in late 2021 to support low-band 5G. Moreover, the ACMA successfully completed the allocation process for area-wide licenses in the 3.4–4.0 GHz band in remote areas of Australia in 2023.

The deployment of 5G is still in progress, and the current implementation of 5G operates in conjunction with 4G, forming what is known as a non-standalone (NSA) network. However, as network evolution advances, 5G is expected to transition into its own separate standalone (SA) network [6]. According to the latest mobility report from Ericsson in November 2023, it was projected that by the end of 2023, 5G mid-band will be deployed in approximately 30 percent of existing 4G sites worldwide [1].

This research was initiated by the swift worldwide implementation of 5G systems, especially in Australia. It is driven by the potential operation of 5G and beyond devices at high-band frequencies, particularly at 26 GHz [7].

Currently, there is an ongoing allocation of spectra suitable for 5G in Australia’s wider 3.4–4.0 GHz frequency range, and while numerous studies in the literature have explored the biological effects of the sub-6 GHz band [8], there is a research gap in the 5G context, specifically regarding mmWaves. In 5G, mmWave refers to frequencies between 24 and 71 GHz (the two frequency ranges 26 GHz and 28 GHz are included in the millimetre range by convention) [1]. Therefore, this study focuses on designing an exposure system operating in the FR2 band, with particular emphasis on the 5G high-band (i.e., 26 GHz).

### 1.2. Perspectives on Health Effects of Non-Ionising Radiation

Considering the public’s concerns about the deployment of 5G technology using mmWave, it is essential to assess whether there are any potential adverse health consequences associated with the levels found in the environment. Monitoring and regulating EMF exposure have emerged as crucial inquiries in this context [9].

The International Commission on Non-Ionising Radiation Protection (ICNIRP) [10], recommended by the World Health Organisation (WHO) for its expertise and impartiality in guiding on health protection related to non-ionising radiation [11], and the Institute of Electrical and Electronics Engineers (IEEE) [12] have established limits to protect the public against known health effects associated with EM fields exposure.

The Australian Radiation Protection and Nuclear Safety Agency (ARPANSA) is the Australian Government’s primary authority on radiation protection and nuclear safety [13]. ARPANSA has established a standard setting acceptable limits to RF fields within the 100 kHz to 300 GHz frequency range [14]. This standard aligns with the ICNIRP 2020 [10]. Given the anticipation of regular reviews and updates to these guidelines/standards, which will heavily depend on ongoing research, it is crucial to address the existing gap in experimental research in RF exposure studies.

Unfortunately, the limited availability and complexity of suitable exposure systems providing high-power RF output have led most studies to either rely on computational simulations [15,16,17,18] or consider synchrotron exposure sources [19,20]. These studies find it challenging to conduct real-time assessments of RF propagation on live tissues. In addition, it was observed that the radiation emitted by the Australian Synchrotron is low to induce any detrimental health effects on tissues with a high water content, such as the cornea [21].

Given the fast adoption of higher frequencies in the EM spectrum and the lack of experimental data at the 26 GHz band in the current literature, our goal was to develop a replicable and dedicated exposure system capable of exposing a wide range of samples to RF radiation under controlled conditions. The key feature of our proposed exposure system is developing an experimental setup in a well-controlled environment, specifically an anechoic chamber shielded from RF interference. It also allows real-time temperature monitoring and can operate effectively at 26 GHz frequency.

### 1.3. Literature Review: Existing Exposure Systems

It is essential to acknowledge that previous research conducted by other scientists has focused on designing exposure systems for various biological studies. An overview of the existing literature, highlighted in Table 1, illustrates the capabilities of exposure systems that have been previously investigated. The table specifically lists exposure systems designed to support frequencies above 26 GHz.

All the mentioned studies have addressed the ocular damage induced by RF radiation. However, as Kojima [26] pointed out, direct comparisons of the study results are not feasible due to variations in their exposure methods, such as the antenna’s shape (i.e., radiation pattern) for mmWave delivery and the use of different experimental animals. Consequently, more research is necessary, and exposure systems must be developed to replicate various studies involving different biological tissues to ensure the accuracy of the results. To the best of our knowledge, none of the previously mentioned studies were conducted in an anechoic chamber, or it was not explicitly mentioned in their experimental procedures. Therefore, our exposure system has an advantage in this regard, operating inside an anechoic chamber.

The main objective of our study was to develop an exposure system to explore the impacts of mmWave radiation on diverse samples. This comprehensive guide, outlining both advantages and limitations, serves as a valuable resource for scientists seeking to replicate exposure systems operating at higher frequencies. We aimed to facilitate further bioelectromagnetics research by conducting experiments, enabling direct comparisons with international studies in the field [22,23,24,25,26,27,28]. Additionally, our aim was to introduce a novel feature: a small radiation spot size specifically designed to study localised exposure. Moreover, the introduced temperature rise measurement system presents a highly sensitive procedure for detecting thermal changes and fluctuations in the temperature rise. This paper thoroughly explains the design, development, and operation of an mmWave exposure system operating at 26 GHz. The performance is assessed to confirm the feasibility of the exposure system for biological studies.

## 2. Materials and Methods

The following sections will elucidate the design, development, and technical information of our mmWave exposure system.

### 2.1. Design and Development of the RF Exposure System within an Anechoic Chamber

**Figure 1:**
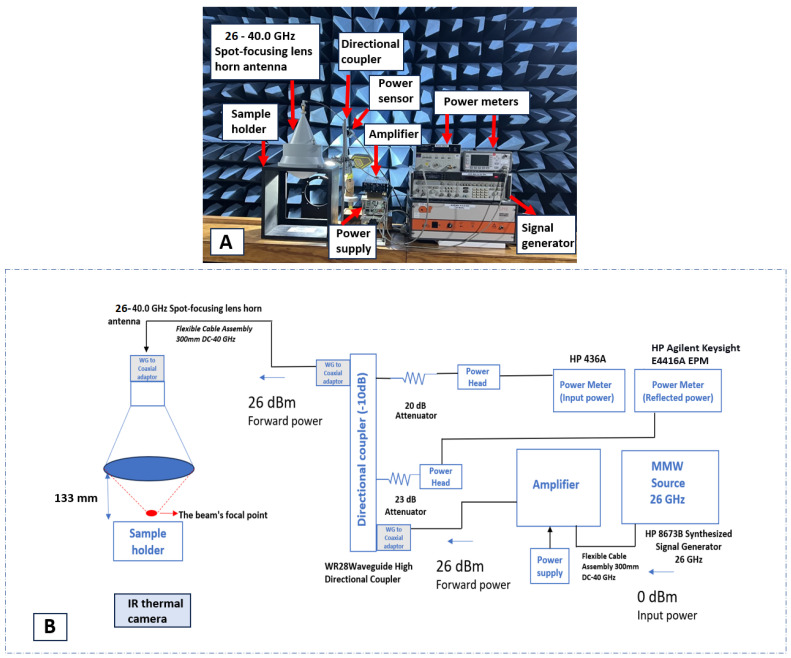
(**A**) The operational mmWave exposure system; (**B**) schematic diagram illustrating the exposure system and its components.

This section provides a detailed description of the designed system and its key RF components (shown in Figure 1A). Figure 1B provides a schematic diagram of the exposure system, representing its RF components. Table 2 offers specific details and specifications of the equipment used in the exposure system.

**Figure 2:**
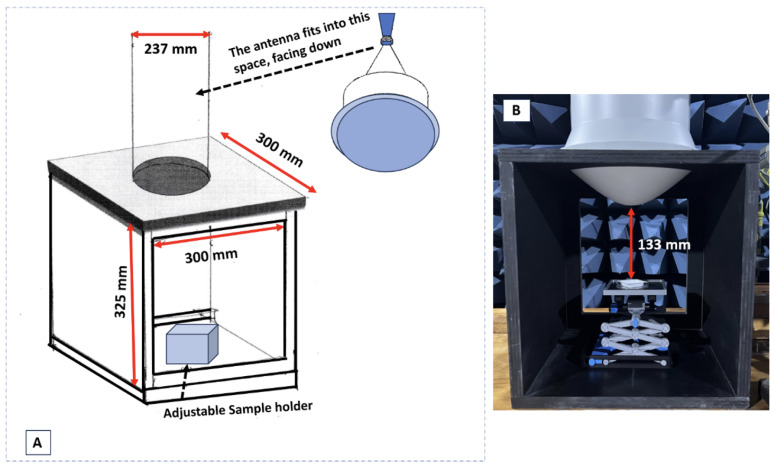
(**A**) Schematic representation of the antenna holder, illustrating its shape and dimensions. (**B**) The antenna holder with the antenna in position and the sample holder being adjusted at a distance of 133 mm from the antenna to capture the focal spot.

The exposure system consists of a spot-focusing lens horn antenna (with a 10 mm focal point). This antenna uses a rectangular waveguide and is linearly polarised. We built an antenna holder using non-metallic material (i.e., wood) to secure the antenna in place. Figure 2A provides the dimensions of this holder. Specifically, we ensured that the centre of the base, located just below the central point of the ‘bulge’, remains visible. This design facilitates the accurate positioning of the sample holder at the focal point. This antenna holder accommodates both the adjustable sample holder and the sample itself. The antenna is connected to an RF signal generator and amplifier at one end. The 8673B Synthesised Signal Generator is a full-performance synthesizer that generates microwave signals over the 2 to 26 GHz range (4 bands) with 1 to 4 kHz frequency resolution. This generator provides −100 to +8 decibel-milliwatts (dBm) output level to 18 GHz, +3 dBm from 18 to 26 GHz.

Using a highly focused antenna with considerable power provides several advantages for biological studies of mmWave exposure, specifically for local exposure. The distance between the antenna and the sample was set at 133 mm, corresponding to the antenna’s focal spot for this particular antenna and frequency of interest (i.e., 26 GHz). This careful design allows for optimal sample placement within the range of the desired exposure limit and the corresponding electric field (E-field). Forward power settings were read from the power meter (Hewlett-Packard Agilent 436A) connected to the forward port of the directional coupler (the port closest to the antenna).

Reflected power readings were obtained from the power meter (HP Agilent Keysight E4416A) connected to the reverse port of the directional coupler (port furthest away from the antenna). The readings were adjusted to account for the isolation provided by the coupler (10 dB), and the additional attenuators (shown in Figure 1B) were inserted to bring the forward/reflected power readings down to a safe level for the power sensors.

We used two IR thermal imaging cameras with a thermal sensitivity of <0.04${\;}^{\circ }$C for temperature-rise measurements. These IR cameras monitored real-time temperature changes in the sample from the same distance and angle. The details of the IR camera are presented in Table 2. The camera captured thermal images of the sample during RF exposure, offering temperature-rise measurements for specific points on the sample surface. Additionally, a high-resolution camera (GoPro 12) recorded temperature fluctuations from the live temperature readings as video. The data obtained from the GoPro were subsequently employed for post-analysis, enabling the monitoring of rapid temperature increases in the samples.

### 2.2. Conducting Measurements in an Anechoic Chamber

RF anechoic chambers are commonly used for measuring antenna radiation patterns, radar cross-sections, and assessing the EM compatibility of diverse equipment. An anechoic chamber is a shielded room created to mimic free space by blocking external RF waves and reflecting off signals from walls, ceiling, or the floor inside the chamber using absorbers. This controlled setting ensures that external RF fields or interference do not influence the measurements, allowing consistent and repeatable results.

Using the wave impedance of free space calculated in Equation (1), the optimal absorber adheres to Equation (2) [31].

(1) $$z_{0}=\frac{E}{H}=\sqrt{\frac{\mu_{0}}{\varepsilon_{0}}}\approx 377\;\Omega$$

where $\mu_{0}=4\pi \times 10^{-7}\;H\cdot m^{-1}$ and $\varepsilon_{0}=10^{-9}/\left(36\pi \right)\;F\cdot m^{-1}$ are the permeability and the permittivity of free space, respectively, and E and H are the magnitudes of the electric and magnetic fields. Perfect impedance matching can be realised if the electric permittivity and the magnetic permeability are equal when the reflection coefficient Γ is zero.

(2) $$\Gamma =\frac{\frac{z_{M}}{z_{0}}-1}{\frac{z_{M}}{z_{0}}+1}=0$$

where $Z_{M}$ is the wave impedance of the absorber. It is important to highlight that, in this specific experiment involving mmWave radiation and a spot-focusing antenna, the anticipated outcome is that the fluid in the dish will absorb the majority of the generated RF.

**Figure 3:**
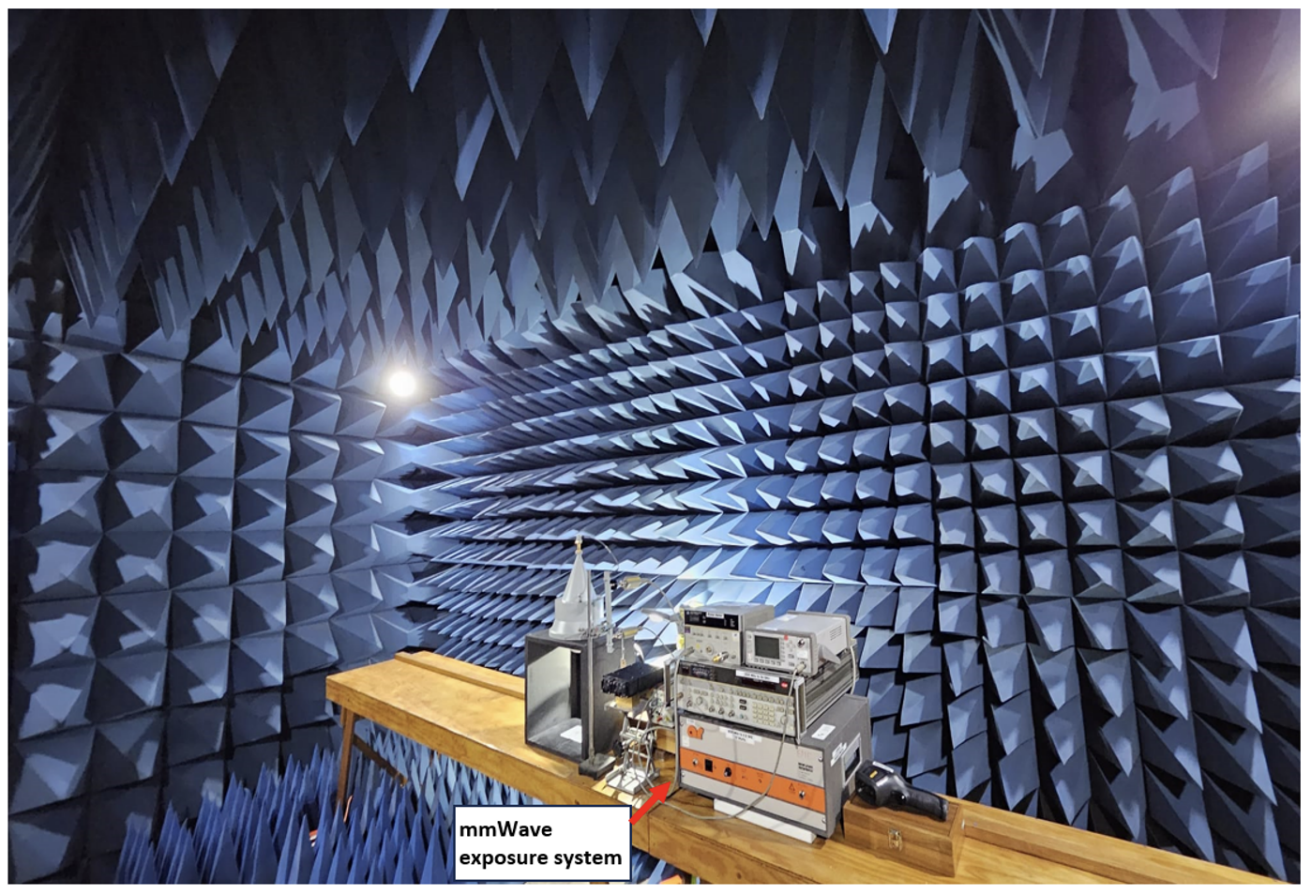
Anechoic chamber facility at the 6G Research and Innovation Lab, Swinburne University of Technology, with the operational mmWave exposure system inside the chamber.

The exposure procedures for this study were carried out in the RF anechoic chamber located at the 6G Research and Innovation Lab, Swinburne University of Technology. The walls of this anechoic chamber are equipped with pyramidal absorbers, as illustrated in Figure 3. The reduction of external and unwanted signals entering our anechoic chamber is achieved by shielding the chamber’s exterior with metal, reflecting noise signals. Utilising an anechoic chamber enhances its efficacy by significantly minimising external interference. Therefore, conducting experiments within this chamber establishes an ideal and controlled environment for studies involving RF frequencies.

### 2.3. Refining Setup for Optimal Radiation Focal Spot

**Figure 4:**
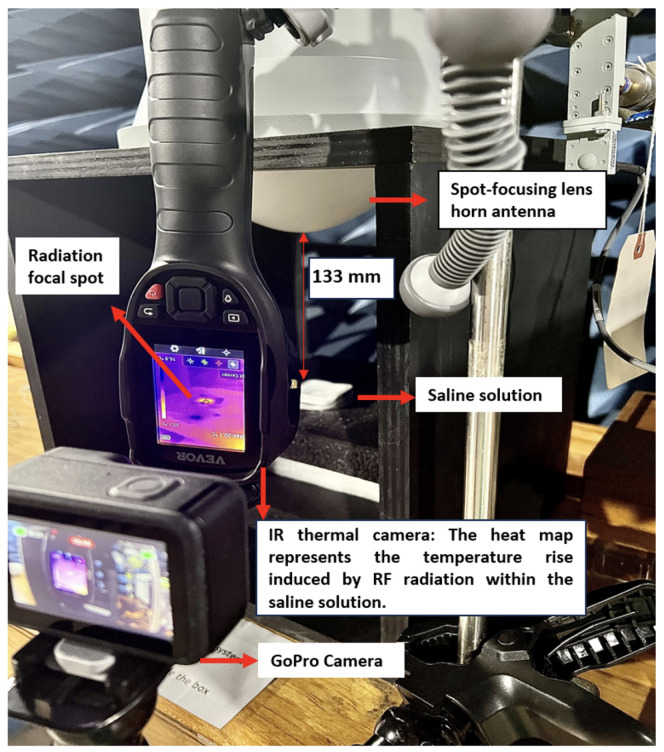
A disposable weighing boat dish filled with saline solution, alongside its corresponding thermal image depicting temperature rise when exposed to 26 GHz radiation.

A paper tissue soaked in saline solution (Sodium Chloride 0.9%), closely mimicking human tissue properties, was positioned in front of the antenna at a distance of 133 mm (Figure 2B). Saline solution was employed as a surrogate for human tissue due to its electrical conductivity properties, which exhibit a degree of similarity to those found in human tissues. This step was carried out to ensure precise sample positioning since the antenna’s focal point is where the maximum power is received. This procedure was repeated multiple times until the optimal location was identified on the saline solution’s thermal image (Figure 4). Once the optimal location was determined, the focal spot’s position was marked, and subsequent samples were positioned in the same spot for exposure.

**Figure 5:**
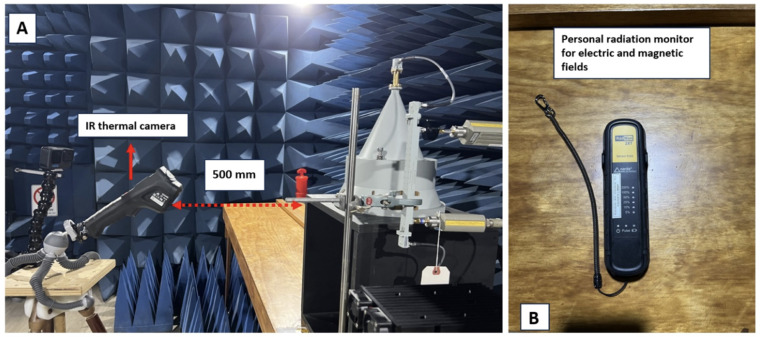
(**A**) Establishing a consistent placement for the IR thermal camera at a fixed distance on the rotatable mount to ensure consistent temperature distribution readings. (**B**) Utilising the 2XT Radiation Monitor, a wearable device for RF-EM field monitoring during the experiment, with frequency ranges for both E-field from 900 kHz to 60 GHz and magnetic (H)-field from 27 MHz to 1 GHz.

Given the high sensitivity of the IR thermal camera to distance variation and movements during temperature measurements, the camera was set at a distance of 500 mm and placed on a tripod at a fixed position to ensure the distance remained constant (Figure 5A). The initial measurement was recorded, followed by subsequent measurements at one-minute intervals. The temperature was measured three times for each interval, and the average was calculated to minimise possible reading uncertainties. A radiation safety monitor (RadMan 2XT) was employed throughout the experiment to ensure the radiation safety was under a safe limit (illustrated in Figure 5B). This wearable device displays the actual field exposure level through LEDs in six steps ranging from 5% to 200%, the safety limit established by the ICNIRP (occupational) [8]. If the field exposure level exceeds the limit value, the device initiates vibration and emits a loud alarm tone signalling for the user to evacuate the area.

**Figure 6:**
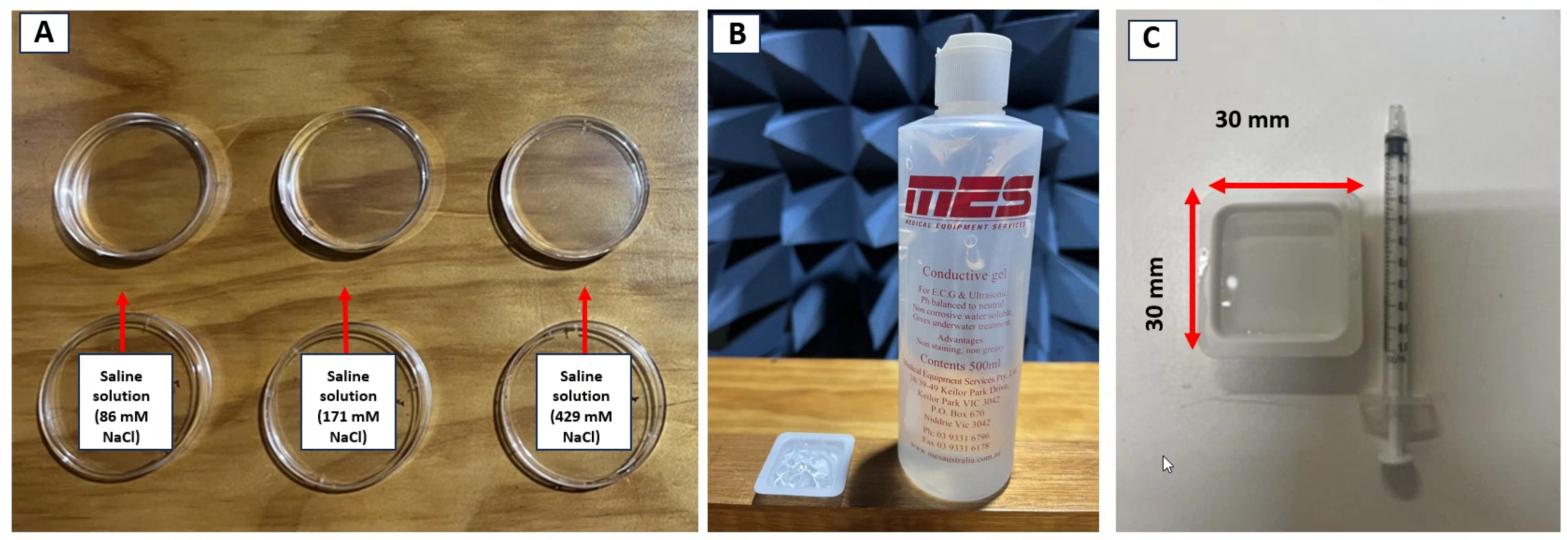
(**A**) Prepared saline solutions with varied NaCl concentrations by mixing distilled water and adding NaCl. (**B**) Conductive gel for ultrasonic and ECG; we transferred 6.5 mL of each solution to disposable weighing boat dishes measuring 30 mm × 30 mm (**C**) using a syringe. These dishes were then placed at the focal spot of the antenna with a spot diameter of 10 mm.

To ensure our experimental design accommodates the unique properties of tissue explicitly designed for biological studies, we considered factors such as sodium chloride (NaCl) concentration in saline solution and the formulation of the conductive gel (the standard gel used for electrocardiogram (ECG) and ultrasound). We used the highly conductive ECG gel to test our hypothesis and evaluate the system’s performance, anticipating its maximum temperature rise. All media were maintained at room temperature (18.3 ± 1.0${\;}^{\circ }$C) and placed in the same disposable weighing boat dish (shown in Figure 6C). The plastic dish used in the experiment had dimensions of 30 mm by 30 mm (length × width) with a depth of 0.5 cm. An identical volume of liquid, precisely measured at 6.5 mL, was employed across all media to ensure consistency.

### 2.4. Media and Solutions Selected for RF Exposure

The major substance in living systems is liquid water, averaging about 75% [21]; while water is an essential component of biological tissues, it is relatively simple compared to the complexity of living tissues. Saline solution is often used as a surrogate in labs and medical settings, closely matching the conductivity of many biological tissues. Pure water consists of two hydrogen atoms and one oxygen atom, while saline water contains various dissolved salts, such as NaCl. Pure water maintains a neutral pH of 7, whereas saline water exhibits a higher pH due to the inclusion of dissolved salts [32]. Before initiating any biological experiments, it is essential to validate the precision of the operating exposure system and its temperature measurement capabilities in accurately representing the thermal characteristics of the samples. To achieve this, we prepared various solutions with varying NaCl concentrations to modulate conductivity. This step allowed us to evaluate the IR camera’s sensitivity in detecting temperature changes associated with the solution’s conductivity alterations.

### 2.5. Saline Solution

The characteristics of saline water closely resemble those of salty water, particularly at lower salinities. The properties of a solution change with increasing dissolved salt content. For instance, as salinity rises, the density of saltwater increases due to the corresponding increase in mass. Additionally, the specific heat capacity decreases with the rise in salinity, as the pure water’s value is higher than that of NaCl under the same conditions. The increase in salinity also results in higher electric conductivity due to the enhanced conductivity of salt ions. To investigate these effects, we exposed saline solutions to RF radiation with varying NaCl concentrations, aiming to understand their impact on temperature rise and explore the effects of the increasing conductivity of the sample.

Saline is a solution comprising water and NaCl, and its concentration can be measured in various ways, such as parts per million (ppm), salinity, weight percent, and molarity (mol/L).

This paper mainly describes the concentration in molarity (i.e., millimolar (mM)), as it provides a more suitable representation for physiological considerations. In Equation (3), *n* is moles of solute (i.e., NaCl with the molar mass of 58.44 g/mol), and *v* is litres of solution.

(3) $$Molarity\;\left(\mathrm{mM}\right)=\frac{n}{v}\times 10^{3}$$

The salinity calculation in percentage (%) is described in Equation (4) where $M_{Salt}$ is the mass of NaCl and $M_{Water}$ is the mass of the solvent (i.e., water), respectively.

(4) $$Salinity\;\left(\%\right)=\frac{M_{Salt}\;\left(g\right)}{M_{Salt}\;\left(g\right)+M_{Water}\;\left(g\right)}\times 100$$

For example, the molarity and salinity calculations for 2.5 g of NaCl with a molar mass of ≈0.0428 and 100 mL of water with a corresponding weight of 100 g are as follows:

$$\begin{array}{c} Molarity\;\left(\mathrm{mM}\right)=\frac{0.0428\;(\mathrm{mol})}{0.1\;(L)}\times 10^{3}\approx 428\;\mathrm{mM}\\ Salinity\;\left(\%\right)=\frac{2.5\;(g)}{100\;(g)+2.5\;(g)}\times 10^{2}\approx 2.44\% \end{array}$$

The same procedure was employed for various NaCl concentrations, as detailed in Table 3. Saline solutions were prepared (displayed in Figure 6) by mixing NaCl and distilled water at room temperature, following the provided calculations.

The thermal conductivity of seawater is approximately 0.58 W/(m·K) [33], while that of pure water is about 0.57 W/(m·K). However, increasing the NaCl concentration in a solution enhances its thermal conductivity [33]. Consequently, adding more salt concentration to water is anticipated to result in a more significant rise in temperature, as the solution would efficiently absorb and conduct EM energy.

#### ECG and Ultrasonic Conductive Gel

The conductive gel for ECG, representing a water-soluble polymer, consists of electrolytes. It exhibits characteristics such as transparency and excellent conductivity. Key specifications for the conductive gel include a pH range of 5.5 to 8 and electrical impedance (*R*) ranging approximately between 13 and 17 Ω [34]. Conductive gels are designed with superior conductivity compared to water, facilitating improved transmission of electrical signals. This enhances the ease of capturing precise and clear signals in various physiological signal acquisition procedures (i.e., ECG). The conductive gel used in this study is a commercial gel designed for ultrasound and ECG applications (shown in Figure 6B).

The composition of the conductive gel used in this paper consists of water (99.05%), carbo-mer (0.55%), imidazolidinyl urea (0.1%), tetrasodium EDTA (0.05%), benzophenones-4 (0.05%), and sodium hydroxide (0.2%).

## 3. Results

In this section, we will present the obtained results. The first part focuses on thermal images acquired using an IR thermal camera, while the second part examines the temperature rise versus time for all the saline solutions and the ECG conductive gel.

### 3.1. Thermal Maps Acquisition through IR Thermal Camera

Using the IR thermal camera shown in Figure 4, we monitored the real-time temperature increase, with thermal images visualising the minimum and maximum temperatures within the media. The sequence of thermal images was captured at 60 s intervals using one camera, while the temperature rise was recorded with another IR camera throughout the entire exposure period. The thermal camera focused on the 1 cm spot of the antenna, capturing the maximum temperature rise. The temperature of the media is indicated in the left corner of each image.

**Figure 7:**
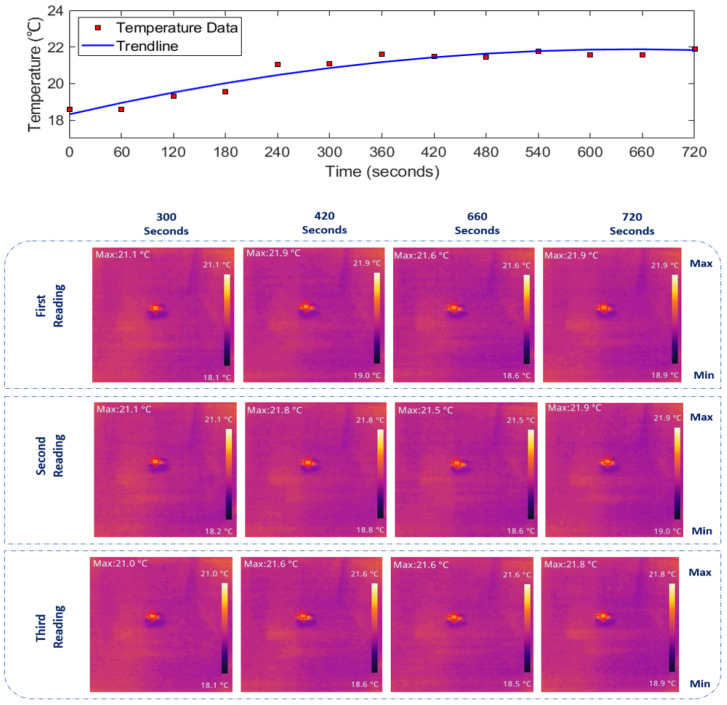
(**Top**): Temperature increase over time. (**Bottom**): IR images depicting the thermal distribution of 86 mM NaCl saline solution exposed to 26 GHz radiation via a spot-focusing lens horn antenna. Concentrated heat at the focal point reflects gradual heating over time, with the corresponding temperature rise shown on the colour scale. Note: Figure 7, Figure 8, Figure 9 and Figure 10: experiments conducted with a forward power of 26 ± 1 dBm (≈400 mW).

**Figure 8:**
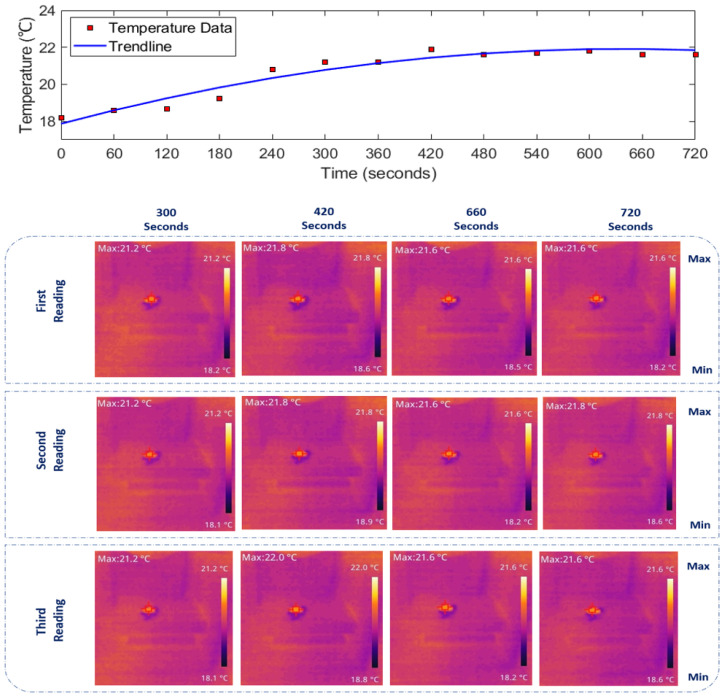
(**Top**): Temperature increase over time. (**Bottom**): IR images depicting the thermal distribution of 171 mM NaCl saline solution exposed to 26 GHz radiation via a spot-focusing lens horn antenna. Concentrated heat at the focal point reflects gradual heating over time, with the corresponding temperature rise shown on the colour scale.

**Figure 9:**
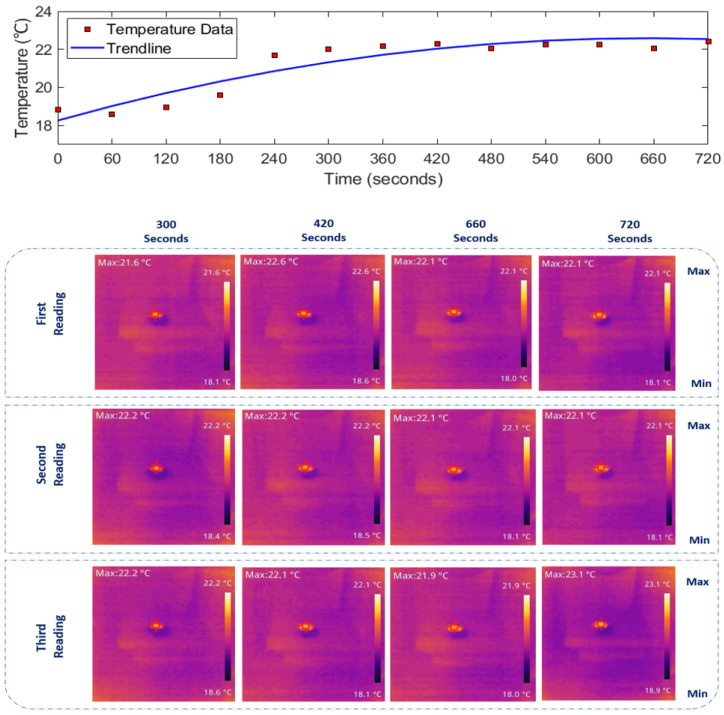
(**Top**): Temperature increase over time. (**Bottom**): IR images depicting the thermal distribution of 428 mM NaCl saline solution exposed to 26 GHz radiation via a spot-focusing lens horn antenna. Concentrated heat at the focal point reflects gradual heating over time, with the corresponding temperature rise shown on the colour scale.

**Figure 10:**
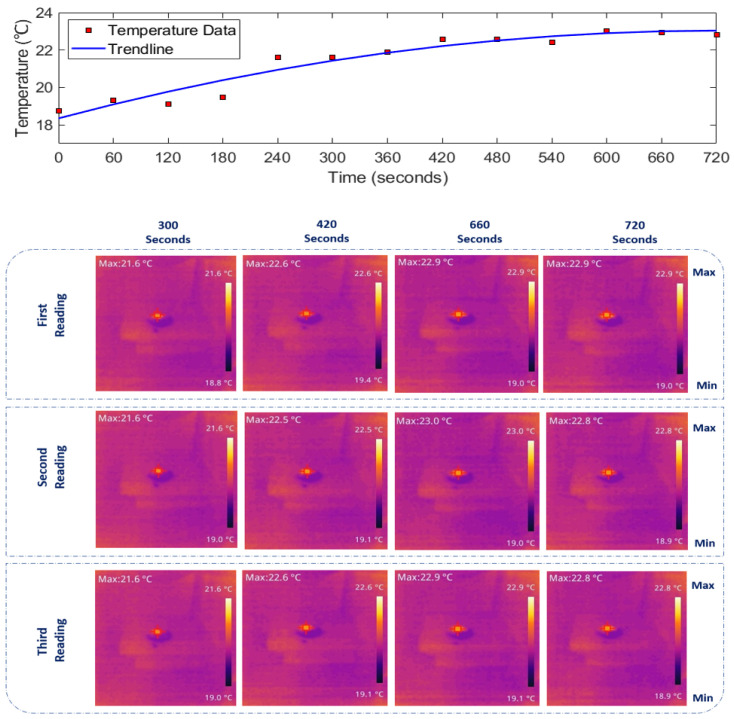
(**Top**): Temperature increase over time. (**Bottom**): IR images depicting the thermal distribution of conductive gel exposed to 26 GHz radiation via a spot-focusing lens horn antenna. Concentrated heat at the focal point reflects gradual heating over time, with the corresponding temperature rise shown on the colour scale.

Given the extensive dataset recorded over approximately 12 min for each medium, including three readings per 60 s, presenting the full results in this paper is impractical. Therefore, specific time data sets (300, 420, 660, and 720 s) have been selected to represent when each medium reached its steady-state temperature. The thermal images depicting temperature rise in Figure 7, Figure 8 and Figure 9 (bottom panels) correspond to saline solutions with NaCl concentrations of 86, 171, and 428 mM, respectively. Similarly, Figure 10 illustrates the thermal image of conductive gel exposed to RF radiation at 26 GHz.

### 3.2. Determining 26 GHz-mmWave Temperature Rise over Time

The top panels of Figure 7, Figure 8, Figure 9 and Figure 10 illustrate the rise in temperature in saline solutions with varying NaCl concentrations and conductive gel over time. By analyzing the data in Figure 7, Figure 8 and Figure 9, we identified the maximum temperature increase in each medium, a topic further explored in the discussion section.

## 4. Discussion

In this section, we will discuss the main findings of the study. The first part highlights verifying the exposure system and examining its accuracy, sensitivity, and feasibility. The second part discusses potential applications for the developed system in the future.

### 4.1. Exposure System Verification

Temperature rise is the primary mechanism for non-ionising radiation exposure [35]; thus, it is crucial to investigate the current system’s proper functioning in generating RF radiation at 26 GHz with a focused beam. Furthermore, it is crucial to ensure that the system’s temperature measurement is precise and responsive, taking into consideration the properties of the sample. Thus, the initial phase of configuring this exposure system is dedicated to ensuring the optimal functionality of the entire setup and the precision of the IR thermal camera’s sensitivity. Once the system’s feasibility is confirmed, the subsequent phase can focus on operating it at various power levels to expose biological samples, including porcine eyeballs, cells, bacteria, etc.

Regarding the system’s operation, the RF equipment in this exposure system functions appropriately in generating RF radiation. The power density at the focal point exhibits dependence on the RF output supplied to the antenna. All experiments were conducted with a forward power of 26 ± 1 dBm (≈400 mW) with an estimated peak power density of 5.1 kW.m^−2^. In the current exposure system setup and design, it is noted that reflected power readings vary based on forward power readings, and notably, they are influenced by the sample being exposed, as well as any absorption or reflection from the sample and the surrounding area (bench, etc.).

As depicted in Figure 7, Figure 8, Figure 9 and Figure 10 in Section 3, the duration of exposure required to observe a noticeable temperature increase is approximately 4 min for all the media and solutions used in this study. The results reveal that in saline solution with moderate conductivity (86 mM NaCl), the temperature increases from 18.6${\;}^{\circ }$C to approximately 21.87${\;}^{\circ }$C. The solution with a NaCl concentration of 428 mM demonstrates a temperature rise starting at 18.83${\;}^{\circ }$C and peaking around 22.43${\;}^{\circ }$C. Across all media, there is a consistent trend of increasing temperature exposed to mmWave radiation. The highest temperature increase is observed in the conductive gel, starting at 18.73${\;}^{\circ }$C and reaching 22.83${\;}^{\circ }$C at the final time point (720 s), suggesting effective absorption and conversion of EM energy. Conductive gels are known for their ability to absorb and dissipate heat efficiently, and this characteristic could contribute to a higher temperature increase when exposed to RF radiation. Furthermore, temperature stabilisation was noted in all media towards the later stages at around 22${\;}^{\circ }$C, indicating a potential saturation (i.e., equilibrium).

The dielectric properties of a material determine its interaction with EMFs [18]. The introduction of NaCl altered the water’s dielectric properties, with a higher concentration of ions changing its permittivity. This modification influenced how the solution absorbed and dissipated EM energy, contributing to an overall increase in temperature. Consequently, the system’s temperature rise measurement accurately considers the properties of the sample, a critical consideration when dealing with biological samples such as the porcine cornea.

The next phase of this research involves exposing porcine eyeballs to 26 GHz and investigating potential damage thresholds. The dielectric properties of water in the 6–300 GHz range result in an absorption coefficient (at 20${\;}^{\circ }$C) rising from 4 cm^−1^ at 6 GHz to 30 cm^−1^ at 30 GHz, 85 cm^−1^ at 100 GHz, and 150 cm^−1^ at 300 GHz [36,37]. The resultant penetration depth at 30 GHz is about 1 mm down to about 0.3 mm at 100 GHz. This feature results in the EM energy being mainly deposited superficially in tissues with a high water content, such as the cornea with 75–80% water [15,38,39]. The depth of penetration of EM fields into the irradiated object decreases as the frequency of the incident field rises [25]; thus, in the eye, higher frequencies penetrate only the cornea’s surface. Indeed, ICNIRP 2020 notes that the part of the eye absorbing very high frequencies (10 to 300 GHz) is the corneal surface [10]. Consequently, a highly precise temperature measurement tool is essential for monitoring temperature changes at the cornea’s surface level. With the system’s feasibility confirmed in this paper, we are confident that the subsequent steps can be appropriately executed.

Another advantage of our designed and developed exposure system is conducting the experiment in an anechoic chamber. Using an anechoic chamber enabled us to make accurate measurements by minimising unwanted reflections and interference, thereby improving the precision and reliability of our data collection. Our anechoic chamber also promotes experimental versatility, offering the flexibility to perform various experiments and tests within a controlled and isolated environment.

### 4.2. Comparison of Exposure Systems

As mentioned previously, the absence of experimental investigations in the 26 GHz band necessitates attention within a controlled environment (i.e., an anechoic chamber). Our literature review indicates that, among exposure systems designed for biological studies, only two studies by Kojima et al. [25] and Sasaki et al. [28] have considered the 26 GHz band. Kojima et al. [25] exposed samples to 18, 22, and 26.5 GHz for 3 min using a K-band system and 26.5, 35, and 40 GHz for 3 min using a Ka-band system. Notably, these studies were not conducted within an anechoic chamber and did not consider the full 26 GHz band, including the range from 25.1 GHz to 27.0 GHz; while the experiments consider various factors associated with mmWave exposure, the authors claim that further research is required to evaluate thermal transport in the eyeballs. For biological investigations, particularly regarding ocular injury, it is crucial for the mmWave exposure system to target incident power precisely at the ocular tissue to prevent temperature elevation in surrounding tissues [28]. Our system achieves a highly localised temperature hot spot (1 cm), even smaller than the reported spot sizes in the existing literature [28]. To the best of our knowledge, none of the previous studies in the literature employed a thermal tracking technology as precise as ours, featuring a screen for real-time temperature tracking with high resolution at the 26 GHz band. Finer details are revealed through high resolution, facilitating the precise identification of temperature changes. The increased number of pixels not only adds more data points but also improves the accuracy of temperature measurements, which is crucial for ensuring the reliability of monitoring temperature elevations. The screen enables real-time observation of temperature variations, while the high resolution ensures detecting and monitoring even the slightest fluctuations. As for our temperature monitoring approach, its superiority lies in the high resolution of the IR thermal camera—240 × 180 pixels. Despite the large number of pixels in the image (43,200), only a small fraction (about 0.21%) relates to the heated sample, highlighting the localised focus of the antenna (1 cm). We focus on approximately 9200 pixels for the heated spot, excluding any potential influence from the surrounding areas. This careful approach ensures accuracy, providing a precise and targeted assessment.

### 4.3. Discussion on Future Applications

As mentioned, establishing mmWave exposure systems is crucial for developing a highly replicable model to study the biological effects of non-ionising radiation, particularly those emitted by 5G devices. Our developed system is specifically designed for biological studies in future research. The observed reflected power and preliminary heating tests suggest the functionality of our designed exposure system. The current system is configured to expose various biological samples to a high-frequency range, commencing at 26 GHz, and is intended for biological studies. With the system’s feasibility now confirmed, the next phase of this research involves using porcine eyeballs to investigate the effects of 26 GHz mmWave on the porcine cornea caused by temperature rises, employing the same exposure setup.

## 5. Conclusions

A mmWave radiation exposure system has been developed within an anechoic chamber, facilitating biological studies of RF radiation exposure at 26 GHz. This versatile radiation source has been effectively used for localised exposure of saline solutions with varying NaCl concentrations and ECG conductive gel. As expected, ions in saline solution enhanced heating mechanisms, a phenomenon thoroughly examined through temperature rise measurements using an IR thermal camera. The temperature monitoring technique employed in this study demonstrates significant sensitivity and precision, essential for conducting thermal effect studies related to RF radiation. The system’s RF reflected power was found to be highly sensitive to sample shape and size at this particular frequency, emphasising the need for consideration in localised temperature rise studies of RF exposure. This well-characterized system, featuring a 1 cm radiation spot size, currently functions at 26 GHz and is feasible for biological studies.

## Figures and Tables

**Table 1:** Existing exposure systems reported in the literature supporting frequencies above 26 GHz. It is important to highlight that the table exclusively includes exposure systems operating beyond 26 GHz, aligning with the primary focus of our research. The table includes information on frequency range, antenna shape, power output, and the technique used for monitoring temperature rise.

Study	Frequency Range	Antenna Shape or Type	Power Output Range	Temperature Measurement	Real-Time Visualisation of Heat Transportation
Khizhnyak et al. [22]	37.5–53.57 GHz and 53.57–78.33 GHz	Horn (round and rectangular) antenna	50 milliwatts (mW)	Infrared (IR) camera	Yes
Kues et al. [23]	60 GHz microwave source	Horn antenna + wave guide	10 mW/cm^2^	IR camera	No
Kojima et al. [24]	162 GHz gyrotron source	Spot-focus-type lens antenna	60–600 mW/cm^2^	Thermography camera	No
Kojima et al. [25]	18–26.5 GHz and 26.5–40 GHz Signal generator	Rectangular horn antenna	200 mW/cm^2^	Thermometer probe, and Microencapsulated thermochromic liquid crystals	Yes
Kojima et al. [26]	40 GHz Signal generator	Lens antenna	200 mW/cm^2^	Microencapsulated thermochromic liquid crystals, IR camera, and fluoroptic thermometer	Yes
Kojima et al. [27]	60 GHz Signal generator	Spot-focus-type lens antenna	200–300 mW/cm^2^ (rabbits with open eye) 400 mW/cm^2^ (rabbits with closed eyes)	IR camera	Yes
Sasaki et al. [28]	26–95 GHz	Spot-focus-type lens antenna	300 mW/cm^2^	Numerical assessment and in vivo	
Kojima et al. [29]	60 GHz	Either a horn antenna or one of the two lens antennas (with diameters of 6 mm and 9 mm).	475 mW/cm^2^ using the horn antenna and 1898 mW/cm^2^ with the lens antenna.	Thermography	No
Ijima et al. [30]	28 GHz	Horn lens antenna (conical)	0–0.0237 mW/cm^2^.	Fiber-optic thermometers and Doppler blood flow meters	Yes

**Table 2:** Detailed information, specifications, and features of the main RF components and equipment employed in configuring the exposure system.

Equipment	Technical Specifications	Frequency Range	Features, Additional Notes, and Comments
Antenna	A-infomw Spot-Focusing Lens Horn Antenna	26–40 GHz	Wave guide: WR28 Polarisation: Linear
Signal generator	HP 8673B Synthesised signal generator	2–26 GHz	Max frequency: 26 GHz Minimum Frequency: 2 GHz Modulation: AM-FM-PULSE Max Output level: 8 dBm Min output level: −100 dBm Resolution: 1 kHz
Amplifier	MI-WAVE 955 Series Power Amplifier	26.5–31 GHz	Gain: 35∼40 dB Psat output: 43 dBm Input power for saturated output: 5–10 dBm Bias: 21 V∼24 V @ 5 A Input/Output: 2.92 mm(F)
Power meter 1	HP 436A	100 kHz–110 GHz	Display readings in Watts, dBm or dB relative eliminating measurement conversion Peaking meter for analogue adjustments Cal factor to compensate each sensor for improved accuracy Power Range: −70 to +44 dBm Power Accuracy: ±0.5% Power Reference: Internal 50 MHz oscillator Type-N output
Power meter 2	HP Agilent Keysight E4416A EPM-P Series Single-Channel	Max. Frequency: depends on power sensor	Channels: 1 Max. power: depends on power sensor Measure: Average Peak
IR thermal imaging camera	VEVOR (Resolution 240 × 180, 2.8′ Screen, −4 to 662${\;}^{\circ }$F temperature range)	N/A	Thermal sensitivity ≤ 0.04${\;}^{\circ }$C

**Table 3:** Various NaCl concentrations with corresponding molarity (mM) and salinity (%).

NaCl (grams)	Molarity (mM)	Salinity (%)
0.5	≈86	≈0.49%
1	≈171	≈0.98%
2.5	≈428	≈2.44%

## Data Availability

Data are contained within the article.
